# Spectrum of Microbial Sequences and a Bacterial Cell Wall Antigen in Primary Demyelination Brain Specimens Obtained from Living Patients

**DOI:** 10.1038/s41598-018-38198-8

**Published:** 2019-02-04

**Authors:** John D. Kriesel, Preetida Bhetariya, Zheng-Ming Wang, David Renner, Cheryl Palmer, Kael F. Fischer

**Affiliations:** 1 Department of Internal Medicine, Division of Infectious Diseases, University of Utah School of Medicine, Salt Lake City, Utah 84132 USA; 2 Department of Pediatrics, University of Utah School of Medicine, Salt Lake City, Utah 84132 USA; 3 Department of Neurology, University of Utah School of Medicine, Salt Lake City, Utah 84132 USA; 4 Department of Pathology, University of Utah School of Medicine, Salt Lake City, Utah 84132 USA; 5 uBiota LLC, Salt Lake City, Utah 84103 USA

**Keywords:** Translational research, Multiple sclerosis, Infection

## Abstract

Multiple sclerosis (MS) is an autoimmune disease characterized by multiple lesions in the brain and spinal cord. We used RNA sequencing to identify microbial sequences and characterize human gene expression patterns in 30 human brain biopsy specimens. RNAs which aligned to known microbial taxa, were significantly enriched in 10 of 12 primary demyelination (MS) brain specimens compared to a group of 15 epilepsy controls, leading to a list of 29 MS microbial candidate genera from 11 different phyla. Most of the candidate MS microbes are anaerobic bacteria. While there were some shared candidates, each of the 10 MS samples with significant microbial RNA enrichment had a distinct set microbial candidates. The fraction of microbial sequencing reads was greater for the MS group (128.8 PPM) compared to the controls (77.4 PPM, p = 0.016). Bacterial peptidoglycan was demonstrated in brain tissue sections from several MS subjects. Human gene expression analysis showed increased expression of inflammation-related pathways in the MS group. This data shows that demyelinating brain lesions are associated with the presence of microbial RNA sequences and bacterial antigen. This suggests that MS is triggered by the presence of a diverse set of microbes within a lesion.

## Introduction

MS is a chronic demyelinating disease of unknown cause, which affects the brain and spinal cord of about 400,000 individuals in the U.S. A number of infections of the central nervous system (CNS) can lead to demyelination, including distemper (dogs), measles (SSPE, humans), JC virus (humans), and influenza (humans)^1^. Microbes, particularly viruses, have long been suspected as causative agents of MS, based on the epidemiology of the disease including geographic patterns, isolated outbreaks, and migration studies^2–5^.

Acute tumefactive MS is an acute tumor-like variant where some patients with demyelinating disease present with large acute lesions, often associated with edema and/or ring enhancement on imaging studies^6,7^. This type of inflammatory demyelinating disease is also called pseudotumoral MS, transitional sclerosis, diffuse myelinoclastic sclerosis, and Marburg variant MS. The initial description by Kepes^6^ suggested that only a few such patients would go on to develop MS. However, a more recent, much larger study of 168 patients with biopsy-confirmed CNS inflammatory demyelinating disease showed that the majority of such patients (79%) go on to develop clinically definite MS^7^. Clinically isolated syndrome (CIS) refers to a single attack compatible with MS, such as optic neuritis. Sixty to 80 percent of patients with a CIS and magnetic resonance imaging (MRI) lesions go on to develop MS, while approximately 20–40 percent have a self-limited process^8–10^.

The pathology of MS is well summarized by Lucchinetti^11^: “The pathologic hallmark of multiple sclerosis (MS) is multiple focal areas of myelin loss within the CNS called plaques or lesions…. Acute active MS lesions are hypercellular demyelinated plaques massively infiltrated by macrophages evenly distributed throughout the lesion forming the classic ‘sea of macrophages.’ These macrophages contain myelin debris, an indication that they have taken up and degraded the remnants of the destroyed myelin sheaths (i.e., active demyelination)”.

Given these factors, including known infectious causes of demyelination and the macrophage-dominated pathology of MS plaques, we considered the possibility that microbes within brain parenchyma might trigger the onset of MS, or the worsening of existing MS disease. In the present study, we hypothesized that the microbial sequence content of primary demyelination brain samples would differ from that in a set of controls. An IRB protocol was written and approved for the collection and analysis of leftover CSF and formalin-fixed paraffin-embedded (FFPE) brain tissue. The feasibility of RNA extractions and deep sequencing from such tissues was demonstrated. This present study involved neuropathology, metatranscriptomics, infectious diseases, and clinical neurology within our institution.

## Results

### Study Population

The characteristics of the study population are shown in Table 1. Age and sex distributions did not differ significantly between the groups. The brain specimen collections were performed somewhat later in the Control group (median 2012) compared with the MS group (median 2007, p < 0.001). This is attributed to the abundance of primary demyelination specimens (relatively rare) compared with epilepsy surgical controls (more common), leading to a situation where the more recent epilepsy control specimens were enrolled. The Control group had significantly more surgical procedures (median 2) in the month preceding the brain specimen collection compared to the MS group (median 1, p = 0.005). The larger number of surgical procedures is due to mapping procedures performed in the controls prior to excision of the epileptogenic focus.

**Table 1:** Characteristics of the Study Population.

Group-Specimen^a^	Age^b^	Sex	Biopsy Year	Biopsy Site^c^	Neuropathology Reading^d^	Clinical Diagnosis^e^	Oligoclonal Bands^f^	Radiologic Findings^g^	DMT^h^
MS-005	48	F	2010	right parietal WM	primary demyelination consistent with MS	RRMS	negative	multiple new lesions over one year	NTZ, IFNb
MS-014	42	F	2007	cortical NOS, gray and WM	demyelinating process consistent with MS	secondary progressive MS	positive	multiple enhancing lesions and cord involvement	GA, NOV
MS-017	52	F	2010	left frontal WM	primary demyelination consistent with MS	MS, untyped	ND	corpus callosum involvement	GA
MS-019	29	F	2007	left parietal WM	demyelinating process consistent with MS	MS, untyped	ND	periventricular enhancing lesion, 2 other small lesions	DMF
MS-021	54	F	2006	biopsy 1: right frontal, gray and WM biopsy 2: right periventricular WM	1: normal 2: primary demyelination consistent with MS	MS, untyped	positive	optic neuritis, multiple demyelinating lesions, Dawson’s fingers	AZP
MS-052	18	F	2008	right frontal, gray and WM	primary demyelination consistent with MS	MS or ADEM	positive	multifocal enhancing lesions, gray matter involvement	none
MS-053	76	M	2009	right parietal NOS	primary demyelination consistent with MS	MS or ADEM	ND	right parietal WM lesions	none
MS-055	27	F	2005	left parietal WM	primary demyelination consistent with MS	MS, untyped	ND	Multiple T2 lesions in subcortical deep white matter	none
MS-056	26	F	2004	left frontal	primary demyelination consistent with MS	MS, untyped	positive	3 poorly described brain lesions	none
MS-057	25	F	2004	right cerebrum, NOS	primary demyelination consistent with MS	MS, CVA	ND	right encephalomalacia	none
MS-062	29	M	2011	right frontal gray matter	perivascular inflammation	progressive MS	negative	T2 lesions, progression, and leptomeningeal enhancement.	GA, NTZ
OND-003	37	M	2011	insular cortex NOS	chronic encephalitis	ADEM, encephalitis	positive	multiple T2 and flair lesions in WM	RTX
OND-018	66	F	2014	right frontal, gray and WM	chronic encephalitis	chronic encephalitis	negative	multiple WM lesions	none
OND-054	53	F	2005	left frontal WM	ischemic or toxic encephalopathy	anoxic injury	negative	diffusion positive WM lesions, frontal hemorrhage	none
C-035	20	F	2012	L occipital	FCD type 2B	Epilepsy	Not applicable		
C-036	35	F	2010	L temporal	Chaslin’s marginal sclerosis	Epilepsy			
C-038	20	F	2010	Frontal	reactive astrogliosis	Epilepsy			
C-039	29	F	2010	L temporal	Chaslin’s marginal sclerosis	Epilepsy			
C-040	60	F	2013	Temporal	FCD type 1A	Epilepsy			
C-041	31	M	2012	L temporal	FCD type 2A	Epilepsy			
C-042	24	M	2013	Frontal	FCD type 2A	Epilepsy			
C-043	42	F	2013	L frontal	FCD type 2B	Epilepsy			
C-044	32	F	2012	R inferior frontal	FCD type 2B	Epilepsy			
C-045	24	F	2012	Frontal	FCD type 2B	Epilepsy			
C-046	48	M	2010	R temporal	Chaslin’s marginal sclerosis	Epilepsy			
C-047	32	M	2012	R temporal	FCD type 1A	Epilepsy			
C-048	27	M	2012	L temporal	FCD type 2A	Epilepsy			
C-049	36	F	2012	not specified	FCD type 2A	Epilepsy			

^a^MS refers to the primary demyelination group; OND = other neurological disease; C = epilepsy control.

^b^Age in years is reported at the time of specimen collection.

^c^Site of brain tissue collection as specified in the pathology reports. The epilepsy control sites were mainly from cortex. Laterality is provided where available. NOS = not otherwise specified, WM = white matter.

^d^Readings on the MS and OND cases as described by the neuropathologist (Au: Palmer). Clinical pathology reports are summarized from specimens from these groups not available for review, and in all controls. FCD = focal cortical dysplasia.

^e^Assessed from medical records and discussions with treating neurologists; RRMS = relapsing-remitting MS; ADEM = acute disseminated encephalomyelitis; CVA = cerebrovascular accident (stroke).

^f^ND = testing not done.

^g^Brain MRI (and/or CT) findings as reviewed with MS neurologist (Au: Renner) or, if not available for viewing, as reported in the medical record.

^h^Disease modifying therapy (DMT) provided after the diagnostic brain biopsy. Since the biopsies in the MS group were to establish a diagnosis, none of these subjects were on disease modifying therapy at the time of the specimen collection. NTZ = natalizumab; IFNb = interferon-beta; DMF = dimethyl fumarate; GA = glatarimer acetate, NOV = novantrone, AZP = azathioprine, RTX = rituximab.

### Characteristics of the Brain Specimens

All brain specimens used in this study were formalin fixed and paraffin-embedded FFPE. Biopsy sites included a mix of white and gray matter in the MS and other neurologic disease (OND) groups. The control biopsies were taken primarily from cortex (gray matter). Ten of the 12 biopsies from the MS group had neuropathology readings of demyelination consistent with MS. One of the subjects (MS-021) was biopsied twice because the first specimen was nondiagnostic (i.e. normal brain). Another subject (MS-062) with well-established progressive MS had a biopsy consisting primarily of gray matter that showed perivascular inflammation. The 3 subjects in the OND group were initially suspected to have MS based on radiology and clinical findings, but were later reclassified based on the neuropathology of their specimens (i.e. *not* primary demyelination).

### Clinical Findings in the MS and OND Groups

Six of the 11 subjects in the MS group had oligoclonal band (OCB) testing in their record. Among these 4 were positive and 2 were negative. One OND subject (OND-003) also had OCB testing which was positive, despite the ultimate diagnoses of acute disseminated encephalomyelitis (ADEM) and encephalitis, not MS. All 11 subjects in the MS group had disease consistent with multiple sclerosis and 6 received disease modifying therapy (Table 1). There is one fewer MS subject than MS samples because one of the subjects (MS-021) had two brain biopsies performed several months apart.

### Sequencing and Alignments

Among the 32 samples (MS 12, Control 15, OND 3, Blank 2), the total sequencing yield was 1.06–2.96 × 10^8^ high-quality read pairs (HQ pairs). There were no significant differences in the number of HQ pairs between the sample groups. Overall, the quality of the sequencing was high with only 2.6% of the original unfiltered read-pairs discarded from the dataset. The remaining HQ pairs were used for the microbial and human database alignments. Most of the HQ pairs (95.7%) were full length (125 bp). The mean length of the remaining trimmed reads (4.3% of the HQ pairs) was 123.9 bp.

Alignments to the human and microbial databases are described in Table 2. Reads that aligned to the human genome were excluded from the microbial analysis. A total of 216,159 concordantly aligning pairs from all 30 experimental samples mapped with high quality and specificity to single sequences in the panmicrobial database (Table 3). Most of these microbial reads were bacterial. They mapped to predominantly rRNA sequences from the phyla Proteobacteria (50.3%), Actinobacteria (20.3%), Firmicutes (16.2%), Bacteroidetes (4.6%), or other (8.5%). The fraction of microbial reads was greater for the MS group (128.8 PPM) compared to controls (77.4 PPM, p = 0.016).

**Table 2:** Sequencing Alignments Summary. The total number of mapped microbial read-pairs was estimated using single aligned read-pairs as reported by Bowtie 2.0 before filtering by mapping quality (MAPQ).

Group	Mean High Quality Read Pairs	Mean Human Genome Pairs^a^	Mean Microbial Pairs^b^	Microbial Fraction (PPM)^c^
MS (N = 12)	7.19E + 07	2.73E + 07	8924 ± 1292	128.8 ± 21.3*
Control (N = 15)	8.17E + 07	3.78E + 07	6487 ± 1158	77.4 ± 10.3
OND (N = 3)	7.01E + 07	2.89E + 07	3925 ± 798	55.1 ± 3.5

^a^Concordant read pairs mapping to either the human genome/transcriptome or the PhiX internal sequencing control.

^b^Concordant read pairs mapping once to the panmicrobial database ± standard error of the mean (SEM).

^c^Microbial concordant read pairs divided by HQ Read Pairs, multiplied by 10^6^ ± SEM.

*P < 0.05 compared with the control group.

**Table 3:** Taxonomic Distribution of Alignments to the Panmicrobial Database.

Level	Microbial Sequences in Database^a^	Aligned in Any Sample^b^	Overrepresented in ≥ 1 MS sample
GI (sequence)	1,265,518	1439	Not done
Species	10,654	735	165
Genus	1,175	392	84
Family	376	185	42
Order	161	97	23
Class	67	38	14
Phylum	39	22	9

^a^Including bacteria, archaea, fungi, protists, and viruses.

^b^Taxa where ≥1 samples align to ≥1 database sequences (MAPQ value ≥  10).

**Figure 1:**
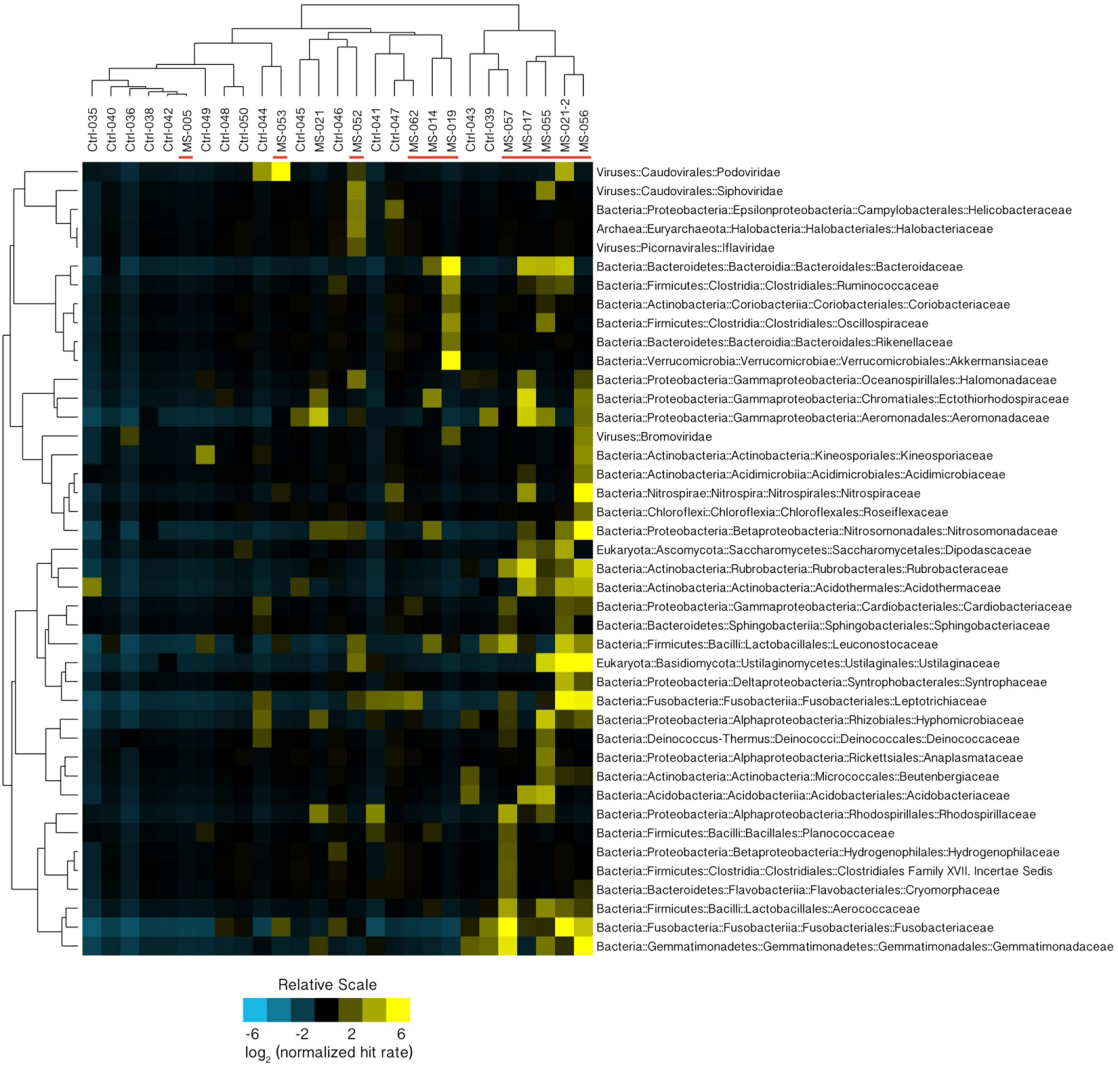
Microbial Families Normalized Hit Rate Hierarchical Cluster 3.0 Analysis^46^. MS candidate microbial families (N = 42) where at least one of the MS samples (red bars) was significantly overrepresented (false discovery rate, q < 0.05) relative to the set of controls is displayed here. The normalized HRs were Log_2_ transformed. Rows were centered by subtracting the mean value for each row from every cell in the row. Yellow indicates an increase over the mean value (black) while blue shows a decrease. On the right of the figure there is a prominent cluster containing 4 MS samples and no controls.

To determine the specific identifies of microbial sequences overrepresented in the MS group, additional filtering was performed using the MAPQ metric, a measure of the specificity of alignment to sequences in the database^12,13^. Human reads, PhiX control reads, and reads where MAPQ < 10 were excluded from further analysis. The remaining alignments have a >90% probability of the reported mapping being to the single best sequence match in the panmicrobial database. Where there is a concordant pair, the probability of a correct mapping is >99%. Significant overrepresentation (q < 0.05) of microbial sequence in at least one of the MS samples was seen for 43 families and 84 genera. The family-level overrepresentation is depicted in Fig. 1. The genus-level overrepresentation is shown in Table 4.

**Table 4:** MS Microbial Candidate List Derived from the Deep Sequencing Data.

Phylum^a^	Family^a^	Genus^a^	MS Mapped Reads^b^	Control Mapped Reads^b^	MS Specimens Increased^c^	Pathogen^d^	Ecology^e^
Proteobacteria	Nitrosomonadaceae	Nitrosospira	5262	0	2	no	Soil bacterium
Actinobacteria	Coriobacteriaceae	Atopobium	3562	90	2	no	Vaginal anaerobic bacterium
Fusobacteria	Fusobacteriaceae	Fusobacterium	3230	56	2	yes	Oral anaerobic bacterium
Basidiomycota	Ustilaginaceae	Ustilago	1668	6	4	no	Corn smut fungus
Proteobacteria	Pasteurellaceae	Aggregatibacter	1166	214	1	yes	Oral anaerobic bacterium
Gemmatimonadetes	Gemmatimonadaceae	Gemmatimonas	848	30	2	no	Soil bacterium
Bacteroidetes	Bacteroidaceae	Bacteroides	834	2	5	yes	Gut anaerobe
Proteobacteria	Nitrosomonadaceae	Nitrosomonas	700	10	2	no	Soil and water bacterium
Fusobacteria	Leptotrichiaceae	Leptotrichia	650	38	2	yes	Oral anaerobic bacterium
Viruses	Podoviridae	Luz24likevirus	590	0	2	no	Bacteriophage (virus)
Verrucomicrobia	Akkermansiaceae	Akkermansia	508	0	1	no	Gut anaerobic bacterium
Firmicutes	Streptococcaceae	Lactococcus	488	6	4	no	Anaerobic fermenting bacterium
Nitrospirae	Nitrospiraceae	Nitrospira	406	4	2	no	Waterborne bacterium
Bacteroidetes	Flavobacteriaceae	Capnocytophaga	358	12	4	yes	Oral anaerobic bacterium
Actinobacteria	Rubrobacteraceae	Rubrobacter	346	2	5	no	Thermophilic bacterium
Actinobacteria	Bifidobacteriaceae	Bifidobacterium	318	52	1	no	Vaginal and gut anaerobic bacterium
Firmicutes	Leuconostocaceae	Leuconostoc	314	30	1	yes	Anaerobic bacterium
Proteobacteria	Moraxellaceae	Psychrobacter	308	50	1	yes	Aerobic bacterium
Firmicutes	Lactobacillaceae	Pediococcus	270	0	1	no	Anaerobic fermenting bacterium
Proteobacteria	Aeromonadaceae	Aeromonas	246	26	2	yes	Waterborne anaerobic bacterium
Proteobacteria	Moraxellaceae	Moraxella	244	18	2	yes	Oral aerobic bacterium
Actinobacteria	Acidothermaceae	Acidothermus	214	36	3	no	Thermophilic bacterium
Proteobacteria	Hyphomicrobiaceae	Hyphomicrobium	196	10	2	no	Soil and water anaerobic bacterium
Proteobacteria	Ectothiorhodospiraceae	Thioalkalivibrio	162	2	4	no	Extremophile bacterium
Firmicutes	Aerococcaceae	Aerococcus	128	2	4	yes	Facultatitive Anaerobic bacterium
Plantomycetes	Planctomycetaceae	Rhodopirellula	120	22	1	no	Marine bacterium
Proteobacteria	Aeromonadaceae	Tolumonas	112	4	3	no	Anaerobic soil bacterium
Firmicutes	Staphylococcaceae	Macrococcus	102	10	2	no	Skin bacterium
Proteobacteria	Rhodospirillaceae	Azospirillum	100	22	1	no	Plant bacterium

In order to qualify as an MS Candidate Microbe at the genus level, at least one specimen from the MS group had read-pair mappings to this taxa significantly increased (q < 0.05) over the control group with MAPQ values ≥ 10 (84 separate genera). The MS microbial genus candidates shown had at least 100 mapped reads among all the specimens in the MS group (29 genera).

^a^As classified in NCBI Taxonomy. Phyla are listed for bacterial and fungal candidates, but not viruses.

^b^Total number of the mapped reads to the taxa derived from the entire MS (N = 12) or Control group (N = 15).

^c^Number of MS specimens (out of 12) where read mappings to this taxa were significantly increased (q < 0.05) compared to the control group (N = 15).

^d^Commonly recognized as a human pathogen.

^e^As described by MicrobeWiki (https://microbewiki.kenyon.edu/index.php/MicrobeWiki), List of Prokaryotic names with Standing in Nomenclature (LPSN, http://www.bacterio.net), UniProt (https://www.uniprot.org), and other online sources.

**Figure 2:**
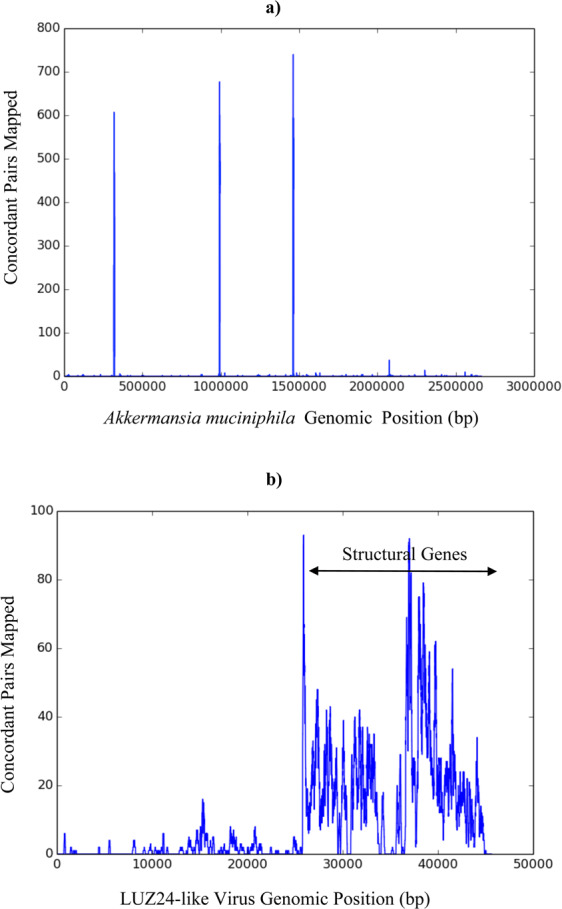
Representative Read-pair Mapping to MS Candidate Microbes. The number of concordant read-pairs mapped (y-axis) to positions along the Akkermansia (**a**) and LUZ24like phage (**b**) genomes (x-axis) are displayed. The mappings shown were done with Bowtie 2.0 alignments without MAPQ filtering. (**a**). *Akkermansia muciniphila* read-pair mapping from MS-019. Peaks at 0.3, 1.0, and 1.5 million bp include sequences that map to 16S and 23S rRNA. (**b**). Pseudomonas phage LUZ24like virus read-pair mapping from MS-053.

Representative mappings of read pair alignments for Akkermansia (sample MS-019) and Pseudomonas phage LUZ24like virus (sample MS-053) are shown in Fig. 2. The distribution of observed RNA abundances across these genomes are consistent with the expected gene expression. For instance, the prokaryote (Akkermansia) mappings are enriched within the rRNA genes with far fewer mappings to other bacterial genes. And for the LUZ24like virus, the observed RNA abundances map to structural genes, as expected in a replicating bacteriophage.

### MS Candidate Microbes

A candidate list of microbes for each MS brain sample was derived by looking for significant outliers within the dataset (see Methods for details of the analysis). Each specimen was compared to the set of controls, with multiple comparisons adjusted using the False Discovery Rate (q < 0.05)^14^. Sequences from 42 microbial families were overrepresented in at least one sample in the MS group. Microbial candidates included 1 archaeal, 35 bacterial, 2 fungal, and 4 viral families (Fig. 1).

Significant overrepresentation (q < 0.05) of microbial sequence in at least one of the MS samples was also seen for 84 genera. (Supplementary data, Genus Level Analysis) This list was filtered to include only those genera where there were 100 or more mapped reads among all members of the MS group. This led to a more tractable list of 29 MS microbial candidate genera from 11 different phyla (Table 4). The candidates with the greatest number of mapped reads in the entire set of MS samples includes the bacterial genera Nitrosospira, Atopobium, Fusobacterium, and Aggregatibacter, and the fungal genus Ustilago, each with more than 1000 mapped reads. The candidates listed in Table 4 include 26 bacterial, 2 fungal, and 1 viral genera. The complete list of 84 genera is available as supplementary data.

Another way of ranking the microbial candidates is by number of specimens significantly increased over controls: Bacteroides and Rubrobacter (5 specimens increased); Ustilago, Lactococcus, Capnocytophaga, Thioalkalivibrio, and Aerococcus (4 specimens increased); and Acidothermus and Tolumonas (3 specimens increased). The MS microbial candidates are also listed by subject (sample). (See Supplementary Data, Subject Specific Microbial Candidates. This data is actually specimen-specific, since subject MS-021 was biopsied twice.) Ten of the 12 MS subjects had at least one overrepresented microbial genus. The number of microbial candidates observed in the samples ranged from 0 to 28.

### Analysis of the MS Cluster

Four MS samples 17, 21–2, 55, 56 had a similar pattern of enrichment of MS candidate microbes at the family level as displayed on the right side of Fig. 1. These samples were compared to the others within the MS group for several technical and clinical parameters. The “cluster” (N = 4) vs. “no cluster” (N = 8) MS subgroups did not differ significantly in total RNA yield, nor in their microbial fractions. All 4 of the cluster samples had macrophage prominence on pathologic analysis, compared to 6 of 8 of the no cluster samples (p = NS). Likewise, the interval between the date of neurologic symptom onset and brain biopsy did not differ between the subgroups. As expected, the cluster group had significantly more microbial candidates at the genus level (mean 21.0) than the no cluster group (mean 6.4, p = 0.01).

### Human Differential Gene Expression

Six hundred eighty-two genes were found to be differentially expressed between the MS and Control groups (FDR < 0.05). Compared to the control samples, the MS samples have many overexpressed immune related genes. Analysis of enrichment by pathway shows 5 immune related pathways, a secretion pathway, and 2 cell-surface interaction pathways are significantly enriched for genes that are overexpressed in the MS samples (Table 5). Conversely the control samples, that are relatively free of macrophages, show relatively higher expression of neuronal genes and enrichment in neuronal pathways (data not shown).

**Table 5:** Human Gene Expression Pathways Overexpressed in MS.

Pathway	Ratio	# Genes in Pathway	# Differentially Expressed Genes: Identity	FDR (q)
Toll-Like Receptors Cascades	0.0208	132	11: IL6R, CD14, LGMN, TLR2, TLR4, TLR7, ITGB2, ITGAM, IRAK3, CTSS, CTSL	<3.33e-04
Trafficking and processing of endosomal TLR	0.0019	12	4: LGMN, TLR7, CTSS, CTSL	2.25e-03
Innate Immune System	0.1046	663	24: C1QC, C1QA, C1QB, FCER1G, IGLC7, WASF2, LYN, IL6R, WIPF1, IGLC2, CD14, LGMN, TLR2, TLR4, TLR7, HLA-E, ITGB2, ITGAM, IRAK3, CTSS, CTSL, TREM2, CD4, TXNIP	3.20e-03
Interferon gamma signaling	0.0114	72	7: CD44, HLA-DRB1, CIITA, HLA-A, HLA-E, IFNGR1, PTAFR	6.00e-03
Endosomal/Vacuolar pathway	0.0014	9	3: HLA-A, HLA-E, CTSS	2.14e-02
Integrin cell surface interactions	0.0103	65	6: CD44, F11R, ITGB2, ITGB1, ITGAM, ITGAX	2.75e-02
Phosphorylation of CD3 and TCR zeta chains	0.0043	27	4: HLA-DRB1, PAG1, CD4, PTPRC	4.04e-02
Platelet Adhesion to exposed collagen	0.0021	13	3: FCER1G, LYN, ITGB1	4.95e-02

Bowtie (v2.2.5.0), tophat (v2.0.14) and cuffdiff (v2.2.1) were used to calculate differential expression levels of known splice variants in the demyelination and control sample groups^12,13^. Pathway enrichment of the differentially expressed genes was calculated with Cytoscape and the Reactome Curated Pathway Database^16^.

### Immunohistochemical Analysis

**Figure 3:**
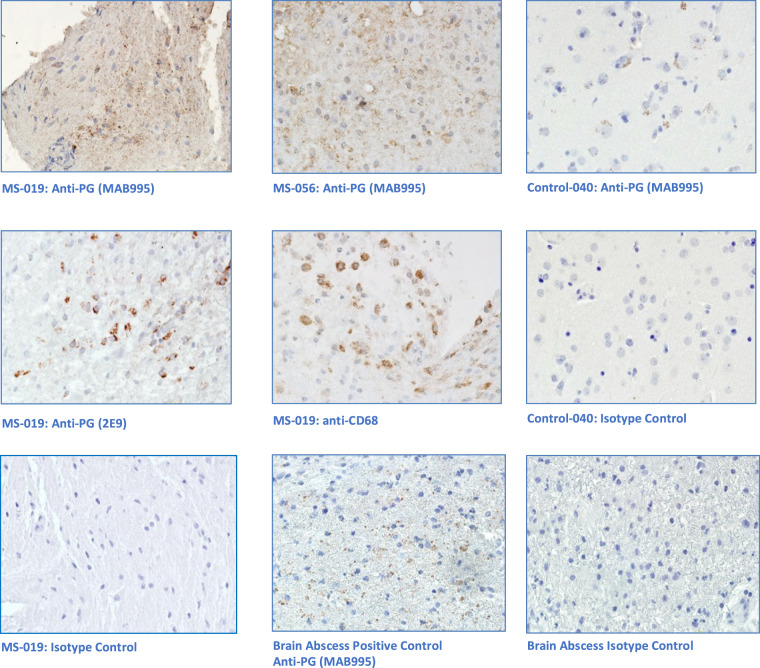
Immunohistochemical Analysis of MS Brain Tissue. Five micron formalin fixed, paraffinized brain tissue sections from four demyelination subjects, three epilepsy controls, and a brain abscess positive control were studied. Photomicrographs displayed are representative. The tissue sections were processed using antigen retrieval followed by casein blocking. The sections were incubated with anti-peptidoglycan mAb (MAB995 or 2E9), anti-CD68 (macrophages, Abcam), anti-lysozyme (macrophages and neutrophils, Abcam), or an equivalent dilution of an isotype control Ab (IgG1, Invitrogen) overnight at 4 degrees. The sections were developed with anti-human IgG-biotin and avidin-HRP. Images shown are all magnified 125x. Brain tissue from subject MS-019 shows specific peptidoglycan staining with two different anti-peptidoglycan mAbs. Peptidoglycan signal is also seen in the brain abscess positive control, specimen MS-056, and epilepsy control subject 040.

Unfortunately, not all the sequenced specimens from the MS group were large enough to allow subsequent immunohistochemical analysis. Four specimens were subjected to staining with antibacterial (peptidoglycan) monoclonal antibodies and controls. Peptidoglycan is a structural cell wall component of both gram-positive and gram-negative bacteria. Representative brain tissue sections from this analysis along with controls are shown in Fig. 3. Peptidoglycan signal was demonstrated in both MS specimens and another brain abscess positive control specimen. Some peptidoglycan signal was also observed in several epilepsy control specimens, but the pattern of staining was much more limited. Matched IgG1 isotype controls support the specificity of the anti-peptidoglycan signal. Anti-CD68 and anti-lysozyme staining confirmed the presence of macrophages and neutrophils in the MS-019 specimen. Conventional neuropathology showed the presence of activated macrophages in most of the other MS specimens.

## Discussion

RNA sequencing and immunohistochemistry both suggest the presence of microbes in many of the MS brain specimens that differed from the controls. In the brain biopsy samples where microbial candidates were detected, sequences mapped to a diverse set of bacterial taxa. Not all the samples shared the same bacterial signatures, and no single bacterial taxon was found in all the samples. This suggests that MS disease is associated with the presence of various microbes within a lesion, or that the macrophage infiltration provides a route for bacterial transport to the lesion. These possibilities are not mutually exclusive and further studies will be required to define how microbial RNA and cell-wall components reach the brain and whether the presence of microbes or their components affects the initiation or course of the disease.

This study has some important limitations. First, the control specimens were taken from tissue affected by epilepsy. Therefore, the control specimens are not from completely normal brain. Autopsy samples could be specifically selected for a normal pathologic appearance, but quality analysis of a few such samples early on in this study showed that the RNA from FFPE autopsy brain samples was of very low quality – too low to be useful for sequencing. Some mismatch between epilepsy controls, taken primarily from cortex (the epileptogenic focus), and the MS samples, containing both cortex and white matter, was therefore unavoidable. We cannot exclude biopsy site effects on the microbial analysis. Second, many of the MS patients received some disease-modifying therapy (DMT in Table 1), while the epilepsy control subjects were treated for their condition with an entirely different set of drugs. We cannot exclude some drug effects on the microbial composition of the samples, although this seems unlikely because neither MS nor epilepsy drugs are considered to be antimicrobials. Lastly, the earlier collection dates among the MS samples compared with the controls probably had little or no effect on the results because there was no discernable relationship between collection date and microbial mapped reads (data not shown).

The authors believe that the data presented here demonstrate a correlation between the presence microbial macromolecules from taxonomically diverse organisms, primarily bacteria, in MS brain lesions. This is supported by distinct mapping of sequencing reads to some bacterial genomes (e.g. Atopobium, Fusobacterium Akkermansia) and by immunohistochemical detection of bacterial peptidoglycan within lesions in several of the subjects. The data reported here does not specifically distinguish between living microbes and the remnants of microbes (e.g. nucleic acid, peptidoglycan) which are no longer viable. However, the presence of the bacteriophage, LUZ24likevirus, in 2 of the MS brain samples (MS-021-2 and MS-053) implies that its host, *Pseudomonas aeruginosa*, was also present. (Pseudomonas itself was likely lost from the final analysis when gram-negative bacterial sequences were filtered out.) Since LUZ24likevirus is a lytic (or virulent), not temperate (or lysogenic), bacteriophage, it is likely that Pseudomonas was also actively replicating in these samples at the time of collection^15^. More work will be required to distinguish replicating from nonreplicating bacteria in the MS brain specimens, but is it possible that nonviable bacterial components may be sufficient to stimulate macrophage infiltration and demyelination.

Microbial RNAs were also observed in the control specimens, and this was also supported by immunohistochemical analysis. The reasons for this are not clear, but might have to do with mapping electrodes placed and open procedures performed in all the epilepsy controls. These open procedures were required to find, map, and remove the epileptogenic foci. Efforts were made to exclude contamination at all points along the pipeline, but the FFPE specimens themselves are necessarily not completely sterile or free of microbial RNA, necessitating the use of an experimentals (MS) vs. controls (epilepsy) study design. Microbial reads in the control specimens might also be telling us something about epileptogenic foci within brain tissue.

Many of the candidate MS microbes (listed in Table 4) are from anaerobic bacterial genera. Many of these might be considered to be commensal bacteria (e.g. Bifidobacterium, Atopobium) and it is currently unclear if they play a role in MS pathogenesis. However, a variety of anaerobic and nonpathogenic bacterial species have also been observed in brain abscesses by sequencing analysis^16^. Since many brain abscess specimens do not grow in culture and the microbiology of these lesions is complex, many or all microbes found within brain abscesses might have some role in the pathologic process. This concept of microbiologic complexity could also apply to MS.

Several of the specimens in the MS group had an enrichment of MS candidate microbes at the family level, shown as a cluster on the right side of Fig. 1. It’s not clear why these 4 samples had a larger number of microbial candidates than the other MS brain biopsy samples.

Laman’s group in the Netherlands first demonstrated bacterial antigen, specifically peptidoglycan, by immunohistochemistry in brain tissue from donors with MS^17^. The authors also demonstrated anti-peptidoglycan antibodies in the CSF of patients with active MS. This group went on to hypothesize that peptidoglycan is involved in the development of CNS autoimmunity^18^. Since that time, a Canadian group led by Chris Power showed bacterial sequence in brain tissue from patients with a variety of CNS diseases, including one patient with MS^19^. More recently, this group performed RNA sequencing on 6 autopsy-derived MS and 6 control brain samples^20^. Their analysis revealed a preponderance of Proteobacteria sequence in the progressive MS and control brain samples, with Actinobacteria sequence predominating in 3 relapsing-remitting MS brain samples. These results were supported by IHC and gene expression studies, and were interpreted as consistent with a disruption of the microbiota within the demyelinating lesions characteristic of MS. The Power group findings are similar to our report here, although we used more controls, deeper sequencing, different mapping and analysis methods, and fixed paraffinized specimens from living subjects.

Enrichment of some bacteria in the stool of MS patients compared with controls has been observed by other groups^21,22^. The study by Jangi *et al*. was also a sequencing study, looking at stool microbiota in MS patients compared with healthy controls. Interestingly, sequences mapped to two genera were increased in the stool of MS patients – Akkermansia and Methanobrevibacter. The present sequencing study in brain tissue specimens, not stool, also identified Akkermansia among the MS candidates (see Table 4).

Subject MS-21 was biopsied twice over the course of several months, acting as its own unintentional control. The first biopsy (MS-21-1) was essentially normal while the second (MS-21-2) showed clear-cut demyelination. Interestingly, only 5 candidate microbes were detected in the first (nearly normal) biopsy with relatively few reads mapped (≤50 each). The second diseased biopsy, however, revealed 28 microbial candidates and 4 of these candidates each had more than 100 reads mapped. While this data is from only a single subject, it does support the hypothesis that demyelinating lesions are associated with more bacteria of multiple types that are significantly different than the controls.

The human gene expression analysis identified Toll-Like Receptors Cascades and Trafficking and processing of endosomal TLR pathways as the most significantly overexpressed in brain tissue among the MS subjects compared to the controls. These pathways include TLR2 and TLR4, both associated with immunity to bacterial pathogens, and TLR 7, associated with immunity to ssRNA (viruses). Other groups have shown an associated between MS and TLRs^23–27^. Interestingly, TLR4 expression is lower in PBMCs from MS patients compared to controls, whereas we detected higher expression in affected brain tissue^24,27^.

The source of the microbial sequences and antigen observed in the MS lesions is currently unknown. There are at least two possibilities: 1) hematogenous seeding from a bacteremia, or 2) microbes were brought in by the infiltrating macrophages. If bacteria are in fact seeded into MS lesions from bacteremia, it seems curious that this has not been discovered before. However, many of the MS candidates identified in this study are anaerobic or even unculturable, and it is well established that recovery of microbes from brain abscesses (with a different pathologic appearance than demyelination) is often difficult. Macrophages could be bringing bacterial RNA and antigens into these MS lesions as a result of an autoimmune process. While macrophages and neutrophils are usually considered to be responders to tissue damage, not initiators, macrophages can actively participate in tissue destruction^28,29^.

Some recent murine studies show that normal gut flora influence brain development and behavior, via peptidoglycan which crosses the blood brain barrier and interacts with pattern-recognition receptor Pglyrp2^30–32^. The source of the peptidoglycan seems to be the developing normal gut flora. Another group studying experimental autoimmune encephalomyelitis (EAE, an animal model disease that resembles MS) showed that peptidoglycan works through receptors NOD1, NOD2, and RIP2 and dendritic cells to worsen the disease^33^. TLR2 and NOD also appear to mediate EAE disease activity in primates^34^. Finally, treatment of EAE mice with oral Lactobacillus paracasei improved measures of disease severity^35^. Whether any of these intriguing findings in mice apply directly to human MS is not clear, but they do demonstrate the importance of peptidoglycan and its sensing molecules to the progression of EAE.

The concept of “immunologic scarring” has been proposed where infections trigger longer term immune dysfunction^36^. This could help explain the persistent or recurrent dysfunction that occurs in MS, even without the persistence of microbial antigen. The present study involved brain biopsies generally taken early on in the disease course for diagnostic purposes.

In the end, the source of the microbial sequence and antigens may be inconsequential. The adaptive immune process seems to be important for the pathogenesis of MS – hence the development of oligoclonal bands representing intrathecal IgG synthesis. It needs to be determined whether the MS microbial candidates identified here are driving a pathogen-specific immune response or not. That is, are there antibodies in the CSF against some of these MS candidate microbes? And, if so, do they account for some of the observed oligoclonal bands seen in most MS patients? These important questions, not answered by the present study, are topics for future investigation.

## Materials and Methods

### Subjects

The study was reviewed and approved by the University of Utah Health Sciences Institutional Review Board (IRB #47316). Informed consent was obtained from all the living subjects or their legally authorized representative. Experiments were performed in accordance with the relevant guidelines and regulations governing human subjects research in the United States.

Characteristics of the study subjects are shown in Table 1. Twelve brain biopsy samples were chosen for sequencing from 11 subjects. Biopsies from 10 of these 11 subjects had pathology showing demyelination. Another subject (MS-062) had well established progressive MS. These 11 subjects are designated, for clarity and brevity, as the MS Group. (One subject, MS-021 was biopsied twice over the course of 3 months.) Findings on the brain biopsies were reviewed by a neuropathologist (Au: Palmer). The disease courses of the MS subjects ranged from a single demyelinating episode to severe fulminant disease (Marburg or tumefactive disease). Clinical information was compiled from electronic records, paper records, and discussions with treating physicians. Clinical diagnoses and imaging findings were reviewed by the MS neurologist in our group (Au: Renner). Three subjects had brain biopsy pathology that showed some other process, designated as Other Neurologic Disease (OND). The Control Group consists of tissue taken from 15 subjects with epilepsy who had some brain tissue removed for control of their seizures. Specimens from the MS and OND subjects were all white matter while those from the epilepsy control subjects were primarily from cerebral cortex (gray matter).

### Specimen Preparation, RNA Extractions, and Library Preparation

The brain biopsy blocks were obtained from the University of Utah Department of Pathology or an outside pathology department (MS-062 only). Multiple procedures were used to prevent exogenous contamination of the samples: a dedicated microtome was used with a fresh blade for each specimen, the bench area was cleaned and decontaminated between processing of specimens, the top 50 microns of tissue was discarded, sterile disposable instruments were used to handle the specimens, and the microtome was cleaned with alcohol and bleach between specimens. Five sections of 10 µm thickness were collected for RNA extractions.

RNA extraction was performed on the brain biopsies using the Qiagen FFPE RNA Kit, which includes a DNAse step. RNA from 12 of these MS brain biopsy specimens passed quality control and all have now been sequenced on the Illumina HiSeq2500 platform in two separate runs. Ten of the 12 sequenced MS samples are from female subjects. One of the female subjects (subject 021) was biopsied twice, 3 months apart. To enrich the samples for microbial sequence, human rRNA was physically depleted from the samples using RiboZero (Illumina Catalog # MRZH116). For sequencing, non-directional complementary DNA libraries were constructed and paired-end 125bp sequencing on a HiSeq-2500 was performed by the University of Utah High Throughput Genomics Core Facility. Fifteen control epilepsy FFPE samples were processed and sequenced in parallel using all the same equipment and procedures. Two chronic encephalitis and one anoxic brain injury samples were sequenced to serve as other neurologic disease (OND) controls. Two blank specimens, defined as no visible tissue (i.e. paraffin only) and <10% of the sample’s reads mapping to human genome or transcriptome, were sequenced and used in the analysis. The RNA-seq yielded 125 bp paired-end reads.

### Sequencing and Quality Control

Quality control of the paired 125 bp reads was performed with the Sickle program. Low-quality pairs were removed and terminal low-quality base calls were trimmed^37^. The Sickle parameters were set to discard read-pairs when either member of the pair was trimmed to <40 bp in length. The HQ read-pairs for each sample were aligned to several databases using Bowtie2 (settings: -q–k1 –phred33–local)^38^. Reads which aligned to the human genome and/or human transcriptome (GRCh37 assembly) were analyzed separately for host gene expression differences. The remaining nonhuman reads were then aligned to a panmicrobial database compiled in the Fischer Lab. The panmicrobial database includes a nonredundant viral database (including complete and partial virus sequences), all complete bacterial, archaea, fungal, and protist genomes in GenBank. This 11 Gb panmicrobial database contains more than 1.3 million sequence records, each identified by a GenBank identifier (gi), representing 10,654 species. Fischer^39^ Microbial alignments were also performed with Bowtie2 (setting: –end-to-end –phred33). Read-pairs aligning to the same microbial sequence (concordant pairs) were counted and carried forward in the analysis.

Since the RNA-seq did not yield exactly the same number of HQ pairs in each sample, the number of reads from a sample that align to each sequence in panmicrobial database were normalized by dividing by the number of reads in high quality pairs (in millions), yielding Pairs Per Million (PPM). These normalized hit rates (HR) were calculated for each microbial taxon and sample. Taxa PPM were aggregated, where possible, and analyzed at the genus and family levels. The data from 2 sequencing runs (Run 1 and Run 2) were combined and analyzed. A Run 2 specific artifact was observed with many more reads aligning to the phylum Proteobacteria than in Run 1. Taxa affected by this artifact were excluded from the HR analysis by comparing the HRs of the Run 2 blank (no tissue) specimens to the HRs of the Run 1 control samples. Then taxa with a HR in one or both blanks greater than the mean HR of that taxa in the controls from sequencing Run 1 were excluded. The blanks were separate FFPE brain biopsy specimens from enrolled subjects that included no visible tissue.

### Human Gene Expression

Bowtie (v2.2.5.0), tophat (v2.0.14) and cuffdiff(v2.2.1) were used to calculate differential expression levels of known splice variants in the demyelination and control sample groups^12,13^. Tophat was run with the ‘—no-novel-juncs’ setting. Cuffdiff was run on the tophat output ‘accepted_hits.bam’ files for each sample. Controlling the false discovery rate (FDR) < 0.05, differentially expressed transcripts were identified between the groups. The transcriptome model used was derived from GRCh37 Ensembl release 75, May 23 2014. Pathway enrichment of the differentially expressed genes was calculated with Cytoscape and the Reactome Curated Pathway Database^40^.

### Statistical Analysis

Demographics and characteristics of the study population were compared using ANOVA, then confirmed with Fishers Exact test for discrete variables (e.g. sex), or the Mann-Whitney nonparametric test (e.g. age, collection year)^41^. A candidate list of microbes for each MS brain sample was derived by looking for significant outliers within the dataset. HRs from each taxon for each sample were log_2_ transformed. Z-scores were calculated for every MS sample using the 15 control samples as the expected distribution. The analysis was performed at the family and genus levels. Z-scores were converted to p-values using the normal distribution. Since we are interested in only in microbial sequence that is over-, not under-represented in the MS group, one-tailed testing was performed. To correct for multiple comparisons, p-values were converted to q-values by the false-discovery rate control method of Benjamini-Hochberg^14^. Candidate MS microbes were defined as those significantly (q < 0.05) over-represented in at least one MS sample compared to the set of 15 controls, *and* with HR > 1.0 PPM. Python 2.7 was used to calculate HR–based significance. HR normalization, transformation and Z-score calculation was performed using numpy and pandas; p- and q- values were calculated with scipy.stats^42–44^.

### Immunohistochemistry

Five micron pathologic brain tissue sections from subjects MS-019 and MS-056, epilepsy controls 039 and 040, and brain abscess (positive control) were studied. The tissue sections were processed using antigen unmasking (Vector Laboratories, Product H3300) followed by 0.5% casein blocking (Sigma). The sections were incubated with anti-peptidoglycan IgG1 mAb (EMD Millipore product MAB995, Temecula, CA), anti-CD68 (macrophages, ab955, Abcam, Cambridge, MA), anti-lysozyme (macrophages and neutrophils, ab108508, Abcam, Cambridge, MA), or a mouse isotype control Ab (IgG1 isotype control, Invitrogen, Catalog Number MA5–14453) overnight at 4 degrees. An additional anti-peptidoglycan monoclonal Ab (mAb 2E9) was kindly provided by Dr. Chris Power, University of Alberta. 2E9 is an IgG3 mAb originally developed in 1994 as a reagent for the detection of intestinal flora-derived bacterial antigen in splenic macrophages^45,46^. This mAb has also been used to show peptidoglycan in brain tissue^17,20^. The sections were developed with biotinylated anti-mouse IgG Antibody (BA-2000, Vector, California, USA) and streptavidin-peroxidase (S5512, Sigma, St. St. Louis, MO, USA). Imaging was performed on a Zeiss Axioplan microscope.

## Supplementary information

Supplementary Information

MS Genus Level Analysis

## Data Availability

The data presented here are freely accessible as Supplementary Data.
