# Supplementary material for: Spectrum of Microbial Sequences and a Bacterial Cell Wall Antigen in Primary Demyelination Brain Specimens Obtained from Living Patients
Source: Sci Rep. 2019 Feb 4;9:1387. doi: 10.1038/s41598-018-38198-8 (PMC6362190; doi:10.1038/s41598-018-38198-8)
Supplement: Supplementary file 1 — Supplementary Information [file 41598_2018_38198_MOESM1_ESM.pdf]

Title: Spectrum of Microbial Sequences and a Bacterial Cell Wall Antigen in Primary Demyelination Brain Specimens Obtained from Living Patients

Authors: John D. Kriesel, Preetida Bhetariya, Zheng-Ming Wang, David Renner, Cheryl Palmer, and Kael F. Fischer

Subject/Sample Specific Microbial Candidates
For all specimens, Taxa (NAME) significantly different than controls with the False Discovery Rate (q) <0.05

MS-005
No significant differences compared to the control group (q all > 0.05)

MS-014

UID	NAME	q-value	Mapped Reads
71245	Dikarya::Ascomycota::Saccharomycetes::Saccharomycetales::Saccharomycetaceae::Kazachstania	5.71E-22	16
106633	::Proteobacteria::Gammaproteobacteria::Chromatiales::Ectothiorhodospiraceae::Thioalkalivibrio	1.21E-10	12
816	::Bacteroidetes::Bacteroidia::Bacteroidales::Bacteroidaceae::Bacteroides	2.25E-10	10
914	::Proteobacteria::Betaproteobacteria::Nitrosomonadales::Nitrosomonadaceae::Nitrosomonas	2.33E-02	12

MS-017

UID	NAME	q-value	Mapped Reads
42255	::Actinobacteria::Rubrobacteria::Rubrobacterales::Rubrobacteraceae::Rubrobacter	5.14E-30	60
816	::Bacteroidetes::Bacteroidia::Bacteroidales::Bacteroidaceae::Bacteroides	1.01E-26	38
106633	::Proteobacteria::Gammaproteobacteria::Chromatiales::Ectothiorhodospiraceae::Thioalkalivibrio	4.46E-26	46
43947	::Proteobacteria::Gammaproteobacteria::Aeromonadales::Aeromonadaceae::Tolumonas	2.91E-15	18
392733	::Acidobacteria::Acidobacteriia::Acidobacteriales::Acidobacteriaceae::Terriglobus	8.15E-13	8
940557	::Acidobacteria::Acidobacteriia::Acidobacteriales::Acidobacteriaceae::Granulicella	8.36E-13	8
2745	::Proteobacteria::Gammaproteobacteria::Oceanospirillales::Halomonadaceae::Halomonas	1.12E-07	8
1865	::Actinobacteria::Actinobacteria::Micromonosporales::Micromonosporaceae::Actinoplanes	9.16E-07	6
449972	::Proteobacteria::Alphaproteobacteria::Rhizobiales::Phyllobacteriaceae::Chelativorans	2.82E-06	6
4951	Dikarya::Ascomycota::Saccharomycetes::Saccharomycetales::Dipodascaceae::Yarrowia	3.78E-05	6
1234	::Nitrospirae::Nitrospira::Nitrospirales::Nitrospiraceae::Nitrospira	7.31E-05	14
963	::Proteobacteria::Betaproteobacteria::Burkholderiales::Oxalobacteraceae::Herbaspirillum	2.88E-04	26
28048	::Actinobacteria::Actinobacteria::Acidothermales::Acidothermaceae::Acidothermus	2.23E-02	26
642	::Proteobacteria::Gammaproteobacteria::Aeromonadales::Aeromonadaceae::Aeromonas	2.53E-02	42
53634	::Actinobacteria::Acidimicrobiia::Acidimicrobiales::Acidimicrobiaceae::Acidimicrobium	3.08E-02	2

MS-019

UID	NAME	q-value	Mapped Reads
239934	::Verrucomicrobia::Verrucomicrobiae::Verrucomicrobiales::Akkermansiaceae::Akkermansia	3.16E-79	254
816	::Bacteroidetes::Bacteroidia::Bacteroidales::Bacteroidaceae::Bacteroides	2.59E-57	270
459786	::Firmicutes::Clostridia::Clostridiales::Oscillospiraceae::Oscillibacter	1.11E-16	14
1263	::Firmicutes::Clostridia::Clostridiales::Ruminococcaceae::Ruminococcus	1.28E-14	12
239759	::Bacteroidetes::Bacteroidia::Bacteroidales::Rikenellaceae::Alistipes	7.13E-10	8
33870	::Actinobacteria::Coriobacteriia::Coriobacteriales::Coriobacteriaceae::Coriobacterium	5.20E-07	6
283168	::Bacteroidetes::Bacteroidia::Bacteroidales::Porphyromonadaceae::Odoribacter	5.29E-07	6
253238	::Firmicutes::Clostridia::Clostridiales::Ruminococcaceae::Ethanoligenens	8.62E-04	4
1506553	::Firmicutes::Clostridia::Clostridiales::Lachnospiraceae::Lachnoclostridium	2.72E-03	30
12316	::::::::Bromoviridae::Ilarvirus	2.03E-02	6

MS-021-1

UID	NAME	q-value	Mapped Reads
364316	::Proteobacteria::Betaproteobacteria::Burkholderiales::Comamonadaceae::Verminephrobacter	3.58E-37	42
43947	::Proteobacteria::Gammaproteobacteria::Aeromonadales::Aeromonadaceae::Tolumonas	6.83E-10	12
106633	::Proteobacteria::Gammaproteobacteria::Chromatiales::Ectothiorhodospiraceae::Thioalkalivibrio	1.59E-06	8
541	::Proteobacteria::Alphaproteobacteria::Sphingomonadales::Sphingomonadaceae::Zymomonas	1.26E-04	4
642	::Proteobacteria::Gammaproteobacteria::Aeromonadales::Aeromonadaceae::Aeromonas	4.33E-02	42

MS-021-2

UID	NAME	q-value	Mapped Reads
5269	Dikarya::Basidiomycota::Ustilaginomycetes::Ustilaginales::Ustilaginaceae::Ustilago	1.80E-46	456
34104	::Fusobacteria::Fusobacteriia::Fusobacteriales::Leptotrichiaceae::Streptobacillus	7.97E-39	36
816	::Bacteroidetes::Bacteroidia::Bacteroidales::Bacteroidaceae::Bacteroides	1.81E-29	42
545932	::::::Caudovirales::Podoviridae::Luz24likevirus	2.38E-29	22
43773	::Proteobacteria::Deltaproteobacteria::Syntrophobacterales::Syntrophaceae::Syntrophus	6.02E-26	18
35798	::Proteobacteria::Betaproteobacteria::Nitrosomonadales::Nitrosomonadaceae::Nitrosospira	1.78E-19	12
1357	::Firmicutes::Bacilli::Lactobacillales::Streptococcaceae::Lactococcus	5.47E-17	22
182639	::Actinobacteria::Actinobacteria::Propionibacteriales::Nocardioidaceae::Kribbella	6.44E-14	8
4951	Dikarya::Ascomycota::Saccharomycetes::Saccharomycetales::Dipodascaceae::Yarrowia	1.87E-13	16

1375	::Firmicutes::Bacilli::Lactobacillales::Aerococcaceae::Aerococcus	6.51E-07	6
253238	::Firmicutes::Clostridia::Clostridiales::Ruminococcaceae::Ethanoligenens	1.54E-06	4
283168	::Bacteroidetes::Bacteroidia::Bacteroidales::Porphyromonadaceae::Odoribacter	1.56E-06	4
929509	::Bacteroidetes::Sphingobacteriia::Sphingobacteriales::Sphingobacteriaceae::Solitalea	1.59E-06	4
42255	::Actinobacteria::Rubrobacteria::Rubrobacterales::Rubrobacteraceae::Rubrobacter	2.85E-06	6
120831	::Bacteroidetes::Cytophagia::Cytophagales::Cytophagaceae::Dyadobacter	7.62E-06	4
1016	::Bacteroidetes::Flavobacteriia::Flavobacteriales::Flavobacteriaceae::Capnocytophaga	2.17E-05	62
697331	::Proteobacteria::Gammaproteobacteria::Pasteurellales::Pasteurellaceae::Basfia	6.36E-04	4
848	::Fusobacteria::Fusobacteriia::Fusobacteriales::Fusobacteriaceae::Fusobacterium	2.88E-03	294
69965	::Firmicutes::Bacilli::Bacillales::Staphylococcaceae::Macrococcus	3.88E-03	10
1243	::Firmicutes::Bacilli::Lactobacillales::Leuconostocaceae::Leuconostoc	8.58E-03	54
32067	::Fusobacteria::Fusobacteriia::Fusobacteriales::Leptotrichiaceae::Leptotrichia	1.06E-02	88
265488	::Planctomycetes::Planctomycetia::Planctomycetales::Planctomycetaceae::Rhodopirellula	1.17E-02	40
869	::Proteobacteria::Gammaproteobacteria::Cardiobacteriales::Cardiobacteriaceae::Dichelobacter	1.66E-02	4
1380	::Actinobacteria::Coriobacteriia::Coriobacteriales::Atopobiaceae::Atopobium	1.80E-02	490
28048	::Actinobacteria::Actinobacteria::Acidothermales::Acidothermaceae::Acidothermus	2.13E-02	24
475	::Proteobacteria::Gammaproteobacteria::Pseudomonadales::Moraxellaceae::Moraxella	2.52E-02	22
416916	::Proteobacteria::Gammaproteobacteria::Pasteurellales::Pasteurellaceae::Aggregatibacter	4.22E-02	190
182709	::Firmicutes::Bacilli::Bacillales::Bacillaceae::Oceanobacillus	4.86E-02	4

MS-052

UID	NAME	q-value	Mapped Reads
1623286	::::::Caudovirales::Siphoviridae::D3112likevirus	4.21E-15	10
2239	::Euryarchaeota::Halobacteria::Halobacteriales::Halobacteriaceae::Halobacterium	2.39E-12	8
441	::Proteobacteria::Alphaproteobacteria::Rhodospirillales::Acetobacteraceae::Gluconobacter	3.76E-09	6
542837	::::::Caudovirales::Podoviridae::Sp6likevirus	1.01E-08	4
2745	::Proteobacteria::Gammaproteobacteria::Oceanospirillales::Halomonadaceae::Halomonas	2.26E-07	8
5269	Dikarya::Basidiomycota::Ustilaginomycetes::Ustilaginales::Ustilaginaceae::Ustilago	1.05E-06	12
232799	::::::Picornavirales::Iflaviridae::Iflavirus	1.95E-05	4
113604	Dikarya::Ascomycota::Saccharomycetes::Saccharomycetales::Saccharomycetaceae::Tetrapisispora	2.45E-05	18
209	::Proteobacteria::Epsilonproteobacteria::Campylobacterales::Helicobacteraceae::Helicobacter	1.06E-02	8

MS-053

UID	NAME	q-value	Mapped Reads
545932	::::::Caudovirales::Podoviridae::Luz24likevirus	2.68E-77	182

MS-055

UID	NAME	q-value	Mapped Reads
940557	::Acidobacteria::Acidobacteriia::Acidobacteriales::Acidobacteriaceae::Granulicella	1.65E-27	24
816	::Bacteroidetes::Bacteroidia::Bacteroidales::Bacteroidaceae::Bacteroides	2.83E-25	38
5269	Dikarya::Basidiomycota::Ustilaginomycetes::Ustilaginales::Ustilaginaceae::Ustilago	1.79E-19	64
1375	::Firmicutes::Bacilli::Lactobacillales::Aerococcaceae::Aerococcus	9.95E-13	14
953	::Proteobacteria::Alphaproteobacteria::Rickettsiales::Anaplasmataceae::Wolbachia	1.25E-11	8
459786	::Firmicutes::Clostridia::Clostridiales::Oscillospiraceae::Oscillibacter	1.28E-11	8
1357	::Firmicutes::Bacilli::Lactobacillales::Streptococcaceae::Lactococcus	2.83E-09	12
81	::Proteobacteria::Alphaproteobacteria::Rhizobiales::Hyphomicrobiaceae::Hyphomicrobium	6.09E-09	46
1263	::Firmicutes::Clostridia::Clostridiales::Ruminococcaceae::Ruminococcus	5.97E-05	4
1298	::Deinococcus-Thermus::Deinococci::Deinococcales::Deinococcaceae::Deinococcus	4.94E-03	6
81682	::Proteobacteria::Betaproteobacteria::Methylophilales::Methylophilaceae::Methylovorus	5.69E-03	6
42255	::Actinobacteria::Rubrobacteria::Rubrobacterales::Rubrobacteraceae::Rubrobacter	9.67E-03	4
497	::Proteobacteria::Gammaproteobacteria::Pseudomonadales::Moraxellaceae::Psychrobacter	9.97E-03	80
4951	Dikarya::Ascomycota::Saccharomycetes::Saccharomycetales::Dipodascaceae::Yarrowia	1.75E-02	4
1016	::Bacteroidetes::Flavobacteriia::Flavobacteriales::Flavobacteriaceae::Capnocytophaga	2.03E-02	22
84756	::Actinobacteria::Actinobacteria::Micrococcales::Beutenbergiaceae::Beutenbergia	2.92E-02	6

MS-056

UID	NAME	q-value	Mapped Reads
35798	::Proteobacteria::Betaproteobacteria::Nitrosomonadales::Nitrosomonadaceae::Nitrosospira	3.50E-155	1746
5269	Dikarya::Basidiomycota::Ustilaginomycetes::Ustilaginales::Ustilaginaceae::Ustilago	3.26E-31	176
1357	::Firmicutes::Bacilli::Lactobacillales::Streptococcaceae::Lactococcus	1.00E-27	60
42255	::Actinobacteria::Rubrobacteria::Rubrobacterales::Rubrobacteraceae::Rubrobacter	9.67E-27	50
374468	Dikarya::Ascomycota::Saccharomycetes::Saccharomycetales::Saccharomycetaceae::Nakaseomyces	1.28E-21	16
53634	::Actinobacteria::Acidimicrobiia::Acidimicrobiales::Acidimicrobiaceae::Acidimicrobium	1.84E-19	8
1234	::Nitrospirae::Nitrospira::Nitrospirales::Nitrospiraceae::Nitrospira	7.87E-17	124

914	::Proteobacteria::Betaproteobacteria::Nitrosomonadales::Nitrosomonadaceae::Nitrosomonas	2.30E-13	208
43947	::Proteobacteria::Gammaproteobacteria::Aeromonadales::Aeromonadaceae::Tolumonas	3.61E-12	14
12300	:::::::Bromoviridae::Bromovirus	3.64E-12	8
34084	::Bacteroidetes::Flavobacteriia::Flavobacteriales::Flavobacteriaceae::Riemerella	5.33E-09	6
120961	::Chloroflexi::Chloroflexia::Chloroflexales::Roseiflexaceae::Roseiflexus	5.43E-09	6
106633	::Proteobacteria::Gammaproteobacteria::Chromatiales::Ectothiorhodospiraceae::Thioalkalivibrio	6.38E-09	10
28065	::Proteobacteria::Betaproteobacteria::Burkholderiales::Comamonadaceae::Rhodoferax	2.52E-05	4
43773	::Proteobacteria::Deltaproteobacteria::Syntrophobacterales::Syntrophaceae::Syntrophus	2.55E-05	4
173479	Gemmatimonadetes::Gemmatimonadetes::Gemmatimonadales::Gemmatimonadaceae::Gemmatimonas	4.50E-05	176
123	::Planctomycetes::Planctomycetia::Planctomycetales::Planctomycetaceae::Pirellula	1.91E-04	10
1873	::Actinobacteria::Actinobacteria::Micromonosporales::Micromonosporaceae::Micromonospora	1.27E-03	4
1817	::Actinobacteria::Actinobacteria::Corynebacteriales::Nocardiaceae::Nocardia	1.65E-03	12
1375	::Firmicutes::Bacilli::Lactobacillales::Aerococcaceae::Aerococcus	2.60E-03	4
1016	::Bacteroidetes::Flavobacteriia::Flavobacteriales::Flavobacteriaceae::Capnocytophaga	2.69E-03	32
32067	::Fusobacteria::Fusobacteriia::Fusobacteriales::Leptotrichiaceae::Leptotrichia	4.91E-03	126
28048	::Actinobacteria::Actinobacteria::Acidothermales::Acidothermaceae::Acidothermus	1.16E-02	32
42054	::Proteobacteria::Gammaproteobacteria::Oceanospirillales::Halomonadaceae::Chromohalobacter	1.96E-02	4
81	::Proteobacteria::Alphaproteobacteria::Rhizobiales::Hyphomicrobiaceae::Hyphomicrobium	3.87E-02	8

MS-057

UID	NAME	q-value	Mapped Reads
1253	::Firmicutes::Bacilli::Lactobacillales::Lactobacillaceae::Pediococcus	1.44E-59	90
1357	::Firmicutes::Bacilli::Lactobacillales::Streptococcaceae::Lactococcus	2.01E-32	74
46255	::Firmicutes::Bacilli::Lactobacillales::Leuconostocaceae::Weissella	2.63E-28	24
1375	::Firmicutes::Bacilli::Lactobacillales::Aerococcaceae::Aerococcus	2.92E-17	18
42255	::Actinobacteria::Rubrobacteria::Rubrobacterales::Rubrobacteraceae::Rubrobacter	1.87E-14	16
33969	::Firmicutes::Bacilli::Lactobacillales::Enterococcaceae::Melissococcus	2.58E-06	4
73918	::Firmicutes::Clostridia::Clostridiales::Clostridiales Family XVII. Incertae Sedis::Thermaerobacter	2.61E-06	4
332102	::Bacteroidetes::Flavobacteriia::Flavobacteriales::Cryomorphaceae::Fluviicola	1.25E-05	4
69965	::Firmicutes::Bacilli::Bacillales::Staphylococcaceae::Macrococcus	1.33E-05	20
848	::Fusobacteria::Fusobacteriia::Fusobacteriales::Fusobacteriaceae::Fusobacterium	8.58E-05	762
1865	::Actinobacteria::Actinobacteria::Micromonosporales::Micromonosporaceae::Actinoplanes	2.26E-04	4

475	::Proteobacteria::Gammaproteobacteria::Pseudomonadales::Moraxellaceae::Moraxella	2.48E-04	56
43988	::Cyanobacteria::::Chroococcales::::Cyanothece	3.54E-04	4
173479	Gemmatimonadetes::Gemmatimonadetes::Gemmatimonadales::Gemmatimonadaceae::Gemmatimonas	1.09E-03	84
919	::Proteobacteria::Betaproteobacteria::Hydrogenophilales::Hydrogenophilaceae::Thiobacillus	5.37E-03	4
1016	::Bacteroidetes::Flavobacteriia::Flavobacteriales::Flavobacteriaceae::Capnocytophaga	6.28E-03	24
648800	::Firmicutes::Bacilli::Bacillales::Planococcaceae::Solibacillus	8.54E-03	4
84567	::Bacteroidetes::Sphingobacteriia::Sphingobacteriales::Sphingobacteriaceae::Pedobacter	1.11E-02	2
1678	::Actinobacteria::Actinobacteria::Bifidobacteriales::Bifidobacteriaceae::Bifidobacterium	1.30E-02	68
1380	::Actinobacteria::Coriobacteriia::Coriobacteriales::Atopobiaceae::Atopobium	1.46E-02	546
869	::Proteobacteria::Gammaproteobacteria::Cardiobacteriales::Cardiobacteriaceae::Dichelobacter	2.06E-02	4
191	::Proteobacteria::Alphaproteobacteria::Rhodospirillales::Rhodospirillaceae::Azospirillum	2.39E-02	18

MS-062

No significant differences compared to the control group (q all > 0.05)

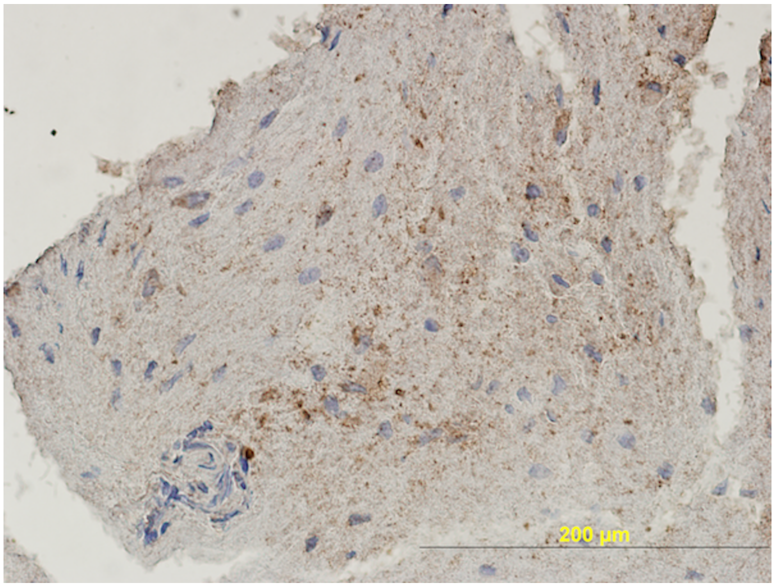
200 µm

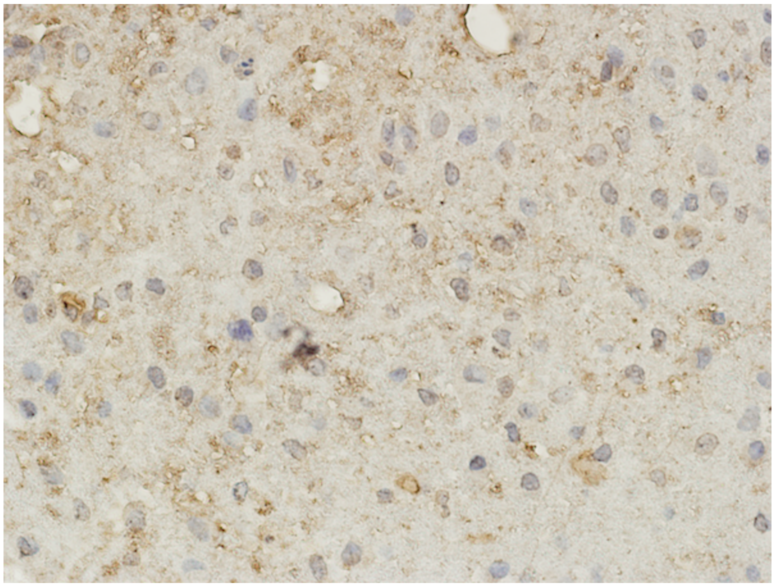

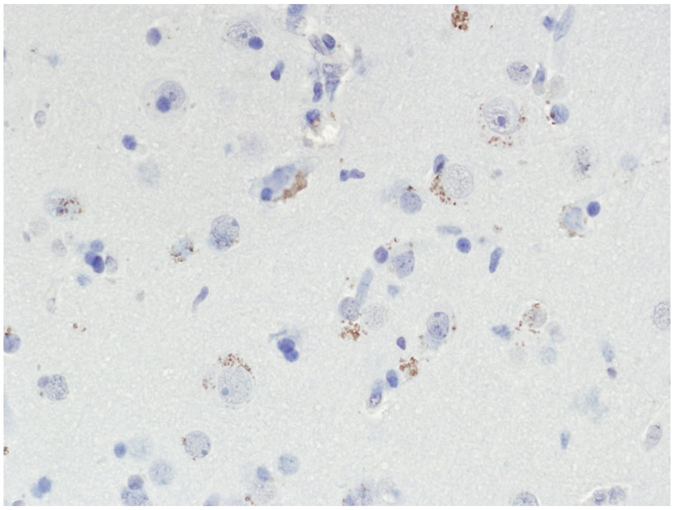

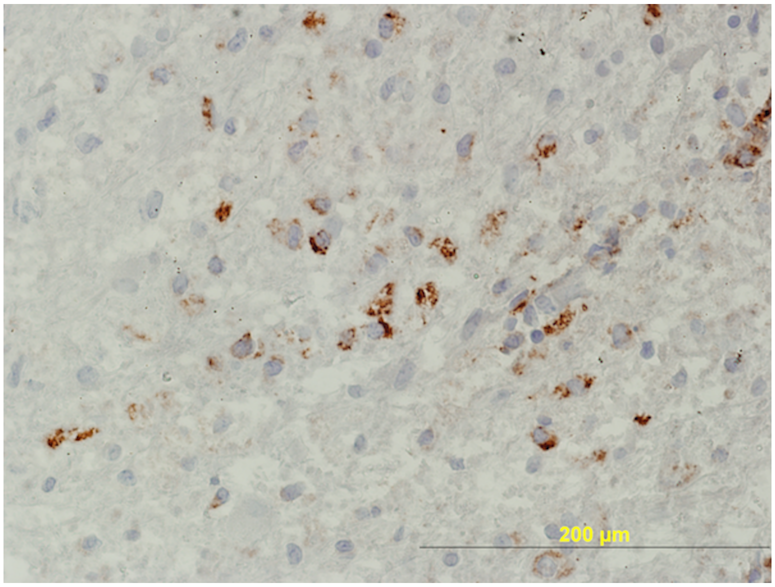
200 µm

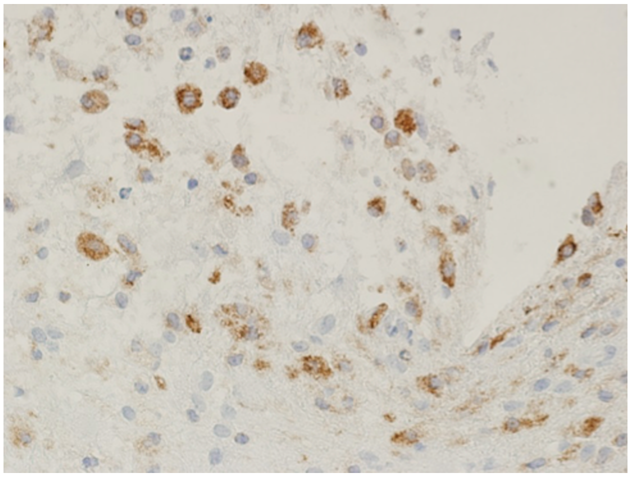

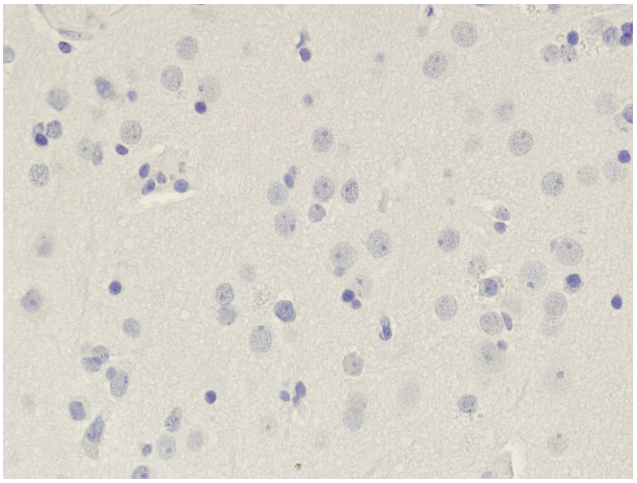

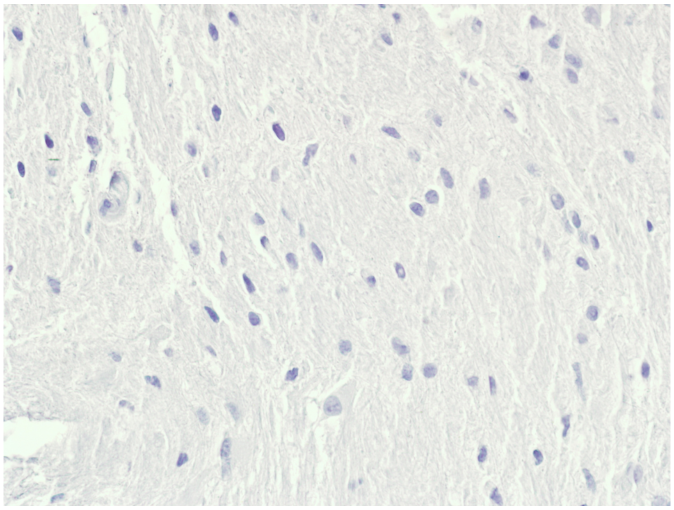

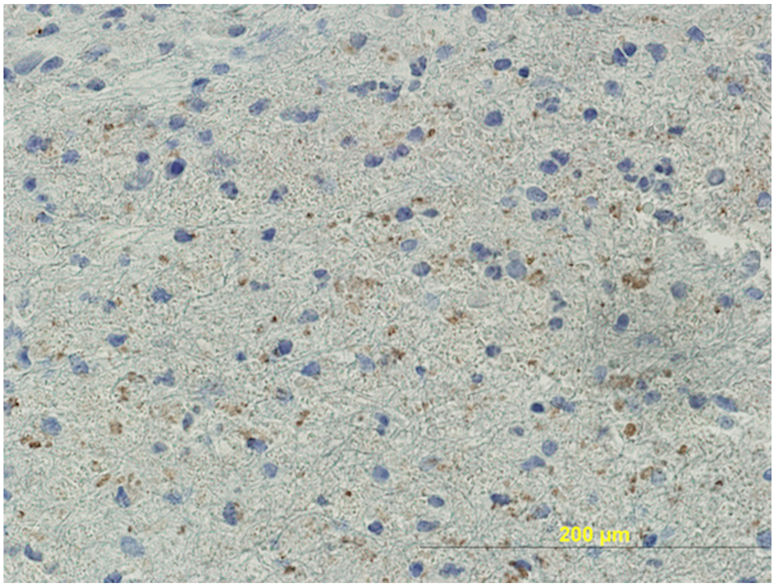
200 µm

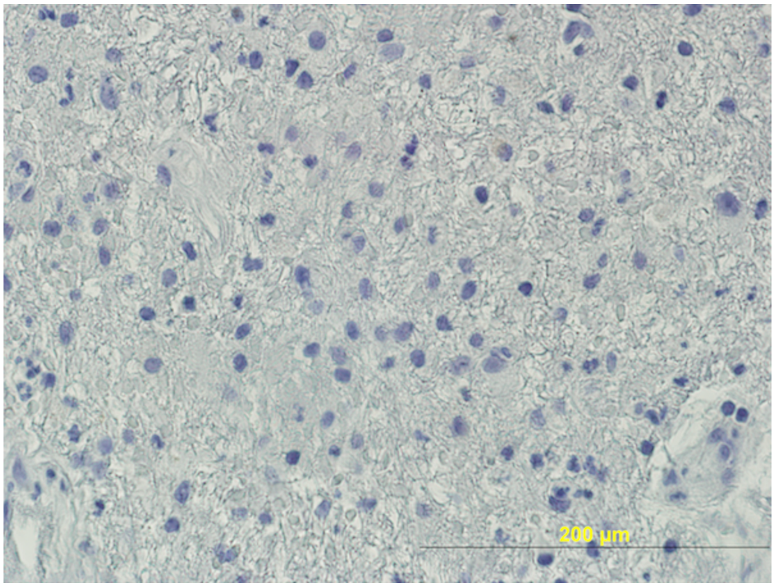
200 µm
